# Monitoring Opioid-Related Social Media Chatter Using Natural Language Processing and Large Language Models: Temporal Analysis

**DOI:** 10.2196/77279

**Published:** 2025-11-04

**Authors:** Grigori Sidorov, Muhammad Ahmad, Pierpaolo Basile, Muhammad Waqas, Rita Orji, Ildar Batyrshin

**Affiliations:** 1 Instituto Politécnico Nacional, Centro de Investigación en Computación, Av. Juan de Dios Bátiz S/N, Nueva Industrial Vallejo, Gustavo A. Madero, Mexico City, 07738, Mexico, 52 5534859107; 2 Dipartimento di Informatica, Università degli Studi di Bari Aldo Moro, Bari, Italy; 3 Department of Software Engineering, The Islamia University of Bahawalpur, Bahawalpur, Pakistan; 4 Faculty of Computer Science, Dalhousie University, Halifax, NS, Canada

**Keywords:** opioid overdose, drug analysis, CDC, Centers for Disease Control and Prevention, large language models, health care, data mining, Reddit, social media, temporal analysis, artificial intelligence, LLM

## Abstract

**Background:**

Opioid overdose is a global public health emergency, with the United States experiencing high rates of morbidity and mortality due to prescription and illicit opioid use. Traditional public health monitoring systems often fail to provide real-time insights, limiting their capacity for early detection and intervention. Social media platforms, especially Reddit, offer a promising alternative for timely toxicovigilance due to the abundance of user-generated, real-time content.

**Objective:**

This study aimed to explore the use of Reddit as a real-time, high-volume source for toxicovigilance and develop an automated system that can classify and analyze opioid-related social media posts to detect behavioral patterns and monitor the evolution of public discourse on opioid use.

**Methods:**

To investigate evolving social media discourse around opioid use, we collected a large-scale dataset from Reddit spanning 6 years, from January 1, 2018, to December 30, 2023. Using a comprehensive opioid lexicon—including formal drug names, street slang, common misspellings, and abbreviations—we filtered relevant posts for further analysis. A subset of these data was manually annotated according to well-defined annotation guidelines into 4 categories: self-misuse*,* external misuse*,* information*,* and unrelated, with distributions of 37.21%, 27.25%, 27.57%, and 7.97%, respectively. To automate the classification of opioid-related chatter, we developed a robust natural language processing pipeline leveraging classical machine learning algorithms, deep learning models, and transformer-based architecture, and fine-tuned a state-of-the-art large language model (LLM; OpenAI GPT-3.5 Turbo). In the final stage, the trained LLM was deployed on an unlabeled dataset comprising 74,975 additional Reddit chatter posts. This enabled a detailed temporal analysis of opioid-related discussions, aligned with 6 years of opioid-related death records from the Centers for Disease Control and Prevention (CDC). For this study, self-misuse and external misuse were merged into a misuse category for direct comparison with the CDC’s mortality data, examining whether trends in social media discourse on opioid misuse reflect patterns in real-world mortality statistics.

**Results:**

The fine-tuned GPT-3.5 Turbo model achieved the highest classification accuracy of 0.93, outperforming the baseline (random forest 0.85) by representing a performance improvement of 9.14% over the machine learning model. The temporal analysis of the unlabeled data revealed evolving trends in opioid-related discussions, indicating shifts in user behavior and overdose-related chatter over time. To quantify this relationship, we calculated the Pearson correlation coefficient between misuse-related posts and CDC death records (*r*=0.854). This correlation was statistically significant (*P*<.001), indicating a strong positive relationship between web-based discussions and CDC mortality data.

**Conclusions:**

This study demonstrates the potential of integrating advanced natural language processing techniques and LLMs with social media data to support real-time public health surveillance. Reddit provides a valuable platform for identifying emerging trends in opioid use and overdose risk. The proposed system offers a proactive tool for researchers, clinicians, and policymakers to better understand and respond to the opioid crisis.

## Introduction

### Background

An opioid overdose occurs when a toxic amount of opioids—such as prescription painkillers like oxycodone, hydrocodone, and morphine or illicit drugs such as heroin and fentanyl—slows vital functions such as breathing and heart rate, potentially leading to death. Opioids can be classified into natural opioids (eg, morphine and codeine), semisynthetic opioids (eg, oxycodone, hydrocodone, and heroin), and synthetic opioids (eg, fentanyl, methadone, and tramadol), each differing in potency, pharmacokinetics, and overdose risk. The United States is currently facing a severe epidemic, with opioids being the leading cause of drug addiction and overdose [1]. In 2017, it was estimated that more than 72,000 deaths occurred due to drug overdoses, and opioids were involved in more than 47,000 of these cases, accounting for approximately 68% of all overdose deaths [2]. This translates to an average of more than 130 opioid-related deaths per day, with approximately 46 deaths daily linked specifically to prescription opioids [3]. The opioid epidemic in the United States has evolved in distinct waves: the first wave was driven by prescription opioids, the second wave was driven by increased heroin use, and the third wave was driven by synthetic opioids, such as fentanyl. Recent reports suggest a potential fourth wave involving polysubstance overdoses, particularly synthetic opioids combined with stimulants [4]. Data from the Centers for Disease Control and Prevention (CDC) highlight that some states, including West Virginia, Ohio, and Pennsylvania, experienced particularly high death rates, exceeding 40 per 100,000 people in 2017, alongside statistically significant year-over-year increases [5].

Variations in opioid overdose mortality across the US states are influenced by multiple factors, including differences in prescription policies and the public’s awareness and education regarding opioid risks and benefits [5]. Despite recognizing these geographic disparities, efforts to monitor the opioid crisis remain severely lacking [6,7]. Existing surveillance systems are hindered by considerable delays, making it difficult to evaluate the immediate impact of new policies and interventions [8-10]. Kolodny and Frieden [11] have highlighted many of the limitations of current monitoring efforts and proposed 10 federal-level actions to combat the opioid epidemic, with enhanced surveillance and monitoring identified as a critical priority.

The US Food and Drug Administration recognizes social media as a valuable source of real-world evidence for drug monitoring [12-14]. In addition to this, social media have increasingly become an important tool for public health surveillance [15-19], offering insights into various health-related trends, including drug abuse and misuse [20-25]. As the use of social media such as X, Reddit, Facebook, and YouTube continues to grow at an unprecedented rate, the volume of health-related discussions shared online has expanded significantly. When analyzed carefully, this chatter offers unique insights into patterns of substance use, which are often difficult to capture through traditional methods. Studies have shown that posts mentioning opioids and other addictive substances often carry clear signs of misuse, with many individuals openly sharing experiences they might hesitate to disclose in clinical settings [26-32]. This behavior highlights a major challenge in addressing the opioid crisis: despite struggling with addiction, many individuals avoid seeking professional help due to barriers such as time constraints, financial hardship, stigma, and a general lack of awareness about available treatment options.

While early studies based on manual analysis highlighted the potential of social media for drug abuse research, fully realizing this potential requires automated, data-driven approaches. However, social media data present a number of challenges for natural language processing (NLP) and machine learning, including frequent misspellings, the use of slang and informal language, data imbalance, and significant noise. To overcome these hurdles, researchers have developed a range of automated techniques, such as rule-based systems, supervised classifiers, and unsupervised learning methods. Notably, most studies exploring opioid-related discussions have relied on unsupervised approaches, often without fully filtering out irrelevant content or rigorously evaluating their methods. Despite these challenges, this body of work has firmly demonstrated the value of social media for toxicovigilance and paved the way for building real-time, end-to-end surveillance systems based on social media data.

NLP plays an essential role in classifying and exploring opioid overdose trends using social media discourse. Social media platforms contain large amounts of first-hand user-generated textual, video, and image data, including posts about drug use, overdose experiences, and discussions on opioid addiction. NLP helps in mining helpful information from these data by identifying keywords and patterns related to opioid use. It also classifies rising overdose cases, tracks geographical trends, and explores public reactions to opioid crises. Techniques such as sentiment analysis, named entity recognition [33], and text classification enable better monitoring. Moreover, NLP can help health care professionals and policymakers by providing real-time insights, helping in early intervention and prevention efforts.

In this study, we designed and evaluated an NLP and a large language model (LLM) to create an automated and robust social media pipeline for toxicovigilance. The proposed system uses supervised LLM to automatically classify opioid-related discussions into 4 predefined categories, such as personal experience, external experience, information, and not related, and integrates temporal data from Reddit to monitor the opioid crisis in specific time frames. For this purpose, we used 6 years of publicly available social media posts from Reddit, dated between January 1, 2018, and December 30, 2023. A total of 5045 posts were manually annotated through a rigorous process, following a set of annotation guidelines specifically developed for this dataset in collaboration with experts in addiction medicine. Each post was categorized into 1 of 4 clearly defined labels. The annotation was performed by 2 domain annotation experts and the corresponding author [34,35]. Final labels were determined through majority voting to ensure consistency and minimize individual bias. Among these, 1877 posts (37.21%) were classified as related to self-misuse, 1375 (27.25%) were classified as related to external misuse, 1391 (27.57%) were classified as informational, and 402 (7.97%) were classified as unrelated. The opioid-related posts were identified using a comprehensive list of prescription and illicit opioid names, including street names.

After preparing the dataset, we applied and evaluated a diverse range of models, including traditional machine learning models, such as logistic regression (LR), random forest (RF), support vector machine (SVM), and Extreme Gradient Boosting (XGB), using term frequency–inverse document frequency (TF-IDF) features. For deep learning, we implemented convolutional neural networks (CNNs) and bidirectional long short-term memory (BiLSTM) networks using pretrained word embeddings, such as FastText and GloVe. In addition, we experimented with 4 transformer-based models—XLM-RoBERTa (XLM-R), DistilBERT, multilingual bidirectional encoder representations from transformers (mBERT), and ClinicalBERT—using contextual embeddings. To further enhance performance, we incorporated a state-of-the-art LLM, GPT-3.5 Turbo, leveraging its contextual understanding for classification. On the basis of our evaluation, the fine-tuned LLM-based model achieved the best performance with an accuracy of 0.93—representing a 9.41% improvement over the best classical machine learning model, RF (0.85). These results highlight the effectiveness of fine-tuning LLMs for capturing nuanced patterns in opioid-related social media discourse.

We analyzed 6 years of social media chatter on the opioid crisis using an LLM, examining 74,975 unlabeled posts from 2018 to 2023. This temporal analysis revealed patterns in online discussions about misuse that often paralleled trends in CDC-reported opioid deaths. A Pearson correlation analysis confirmed a strong positive association between misuse-related posts and CDC death records (*r*=0.854, *P*<.001), highlighting that social media activity closely reflects real-world mortality trends.

### Prior Work

Webster et al [36] analyzed Canadian news media coverage of opioids, highlighting a shift from medical pain treatment to criminalization. They showed how media narratives polarize individuals as either victims or criminals while downplaying the pharmaceutical industry’s role. This framing leads to stigmatization and punitive responses rather than effective solutions.

Dekeseredy et al [37] analyzed 5068 tweets about medications for opioid use disorder to understand public discourse. They found themes of suspicion, conspiracy theories, frustration over treatment access, and stigmatizing language. This stigma on social media may discourage people from seeking treatment.

Russell et al [38] analyzed 2836 Facebook comments on news posts about the opioid epidemic in Ohio. They found 2 main perspectives: most (61.1%) supported addiction as a disease requiring treatment, whereas a significant minority (38.9%) blamed individuals and dismissed the crisis. Media framing did not influence how addiction was discussed. These findings highlight both support and stigma in public discourse.

Hedberg [39] analyzed how male and female opioid users are represented in Ohio newspapers, revealing gendered biases. Male users are portrayed more leniently, often with humor, whereas female users are stigmatized as “erratic” and penalized for failing as caregivers. This language reinforces societal perceptions and may shape policy responses.

Johnston [40] analyzed racial representation in Canadian news media coverage of opioid deaths, highlighting differences in how White and Indigenous users are portrayed. They argued that White users are often seen as innocent victims, whereas Indigenous users face stigma. Drawing on media studies by Hall and others, the author examined how race influences drug-related narratives. They also discussed harm-reduction policies and Indigenous leaders’ calls for culturally based treatment approaches.

Jaafar [41] analyzed language patterns in social media discussions of drug addiction, focusing on common metaphors and frames. Using corpus linguistics, it finds that addiction is largely associated with negative and life-threatening risks. The research highlights how language shapes public attitudes and informs policy and health communication strategies.

Henson [42] analyzed public perceptions of fentanyl through YouTube videos and comments, revealing both informed discussions and illicit drug marketing. Using semantic network analysis, it identifies public awareness of evidence-based treatments and the sources of the crisis. The findings highlight the need for health policy interventions to protect online discourse and combat illegal drug sales.

Sarkar et al [43] created a labeled dataset of 9006 geolocated tweets, categorized into 4 classes: abuse related, informational, unrelated, and non-English, and trained multiple machine learning classifiers, with their best ensemble model achieving a micro-averaged *F*_1_-score of 0.726.

Fodeh et al [44] collected 6988 opioid-related tweets, manually labeled them into 3 categories (No-OM, pain-related OM, and recreational OM), and applied NLP with multiclass machine learning classifiers. Using a held-out test set of 1677 tweets, XLNet achieved the best performance with *F*_1_-scores of 0.71 (pain misuse) and 0.79 (recreational misuse) and macro– and micro*–F*_1_-scores of 0.82 and 0.92, respectively. They also performed k-means clustering on terms, revealing distinct keyword clusters associated with each misuse category.

Ali and LoveJennifer [45] applied a machine learning classifier to identify Twitter users self-reporting nonmedical prescription drug use (NMPDU) and analyzed 137 million posts from 87,718 users using NLP. They found NMPDU users showed more negative and fewer positive emotions, with greater concerns about family and the past and less about work and health compared to controls. Gender analysis revealed females expressed more positive emotions, anticipation, and health concerns than males across all drug categories.

Garg et al [46] manually labeled 391 Reddit posts using a clinical codebook of 12 fentanyl risk categories and trained machine learning models to detect risky content. Their best model achieved 76% accuracy and sensitivity in identifying posts indicative of fentanyl misuse. They also compiled a vocabulary of colloquial terms for fentanyl to aid future monitoring and interventions.

Preiss et al [47] developed a named entity recognition model to extract substances and effects from 4 million Reddit comments discussing opioid withdrawal and built a co-occurrence network to link remedies to symptoms. Their model achieved *F*_1_-scores of 92.1 (substances) and 81.7 (effects), identifying 458 substances and 235 effects, with 41.5% of remedies being US Food and Drug Administration–approved and others potentially useful, natural, or harmful. They created the Withdrawal Remedy Explorer to enable further analysis, validating social media as a method for studying self-treatment behaviors.

Yao et al [48] analyzed Reddit posts to detect suicidal ideation among opioid users using machine learning, training classifiers on data from suicide, depression, opioid-related, and control subreddits. The best model, a CNN, achieved an *F*_1_-score of 96.6%, whereas neural network models outperformed traditional methods in minimizing false negatives when predicting posts combining opioid use and suicidality. This study shows that social media data and neural networks can help identify individuals at high risk for intentional overdose.

Smith et al [49] analyzed Reddit data to monitor opioid trends, creating a cohort of 1,689,039 users assigned to states and tracking opioid-related posts over time. Using an NLP pipeline, they compared Reddit trends with CDC overdose and NFLIS data, finding that adding real-time Reddit data improved the prediction of synthetic opioid overdose rates despite the CDC’s 6-month reporting lag. The study shows Reddit can enhance timely surveillance of the opioid epidemic.

Unlike previous studies, including Sarkar et al [43], which focused on general abuse detection without distinguishing between types of misuse, our work develops a fully supervised NLP pipeline using both traditional machine learning and a fine-tuned LLM (GPT-3.5 Turbo) to classify Reddit posts into 4 detailed categories: self misuse, external misuse, informational, and unrelated. While prior studies often analyzed specific aspects such as pain versus recreational misuse by Fodeh et al [44], NMPDU behaviors by Ali and LoveJennifer [45], or fentanyl risk by Garg et al [46], our approach integrates multiple dimensions, spans 6 years of Reddit data, and incorporates temporal analyses. This enables not only higher classification accuracy (0.93) but also robust, scalable, and temporally informed monitoring of opioid-related social media discourse, providing a more comprehensive toxicovigilance system than previous work.

## Methods

### Construction of Dataset and Preprocessing

To construct a comprehensive dataset for analyzing opioid overdose discussions, we turned to Reddit, one of the most widely used online platforms where users openly share their personal health stories and experiences. Reddit is home to a range of communities, or subreddits, where users discuss various health-related topics, including substance use and recovery. Recognizing the value of real-world, user-generated content, we used Reddit’s public application programming interface in combination with the Pushshift application programming interface to collect a substantial dataset of approximately 80,000 posts. The data, focused on mentions of opioid drugs and their overdose effects, were gathered between January 1, 2018, and December 30, 2023.

The data collection process spanned several relevant subreddits, such as r/opiates, r/OpiatesRecovery, r/addiction, and r/Drugs, which are known for hosting candid discussions about opioid misuse, addiction recovery, and overdose experiences. These subreddits provided a diverse range of conversations, from individuals sharing their personal struggles with addiction to those discussing family members’ or friends’ overdose incidents, as well as general awareness and information about opioids and related substances.

**Figure 1.**
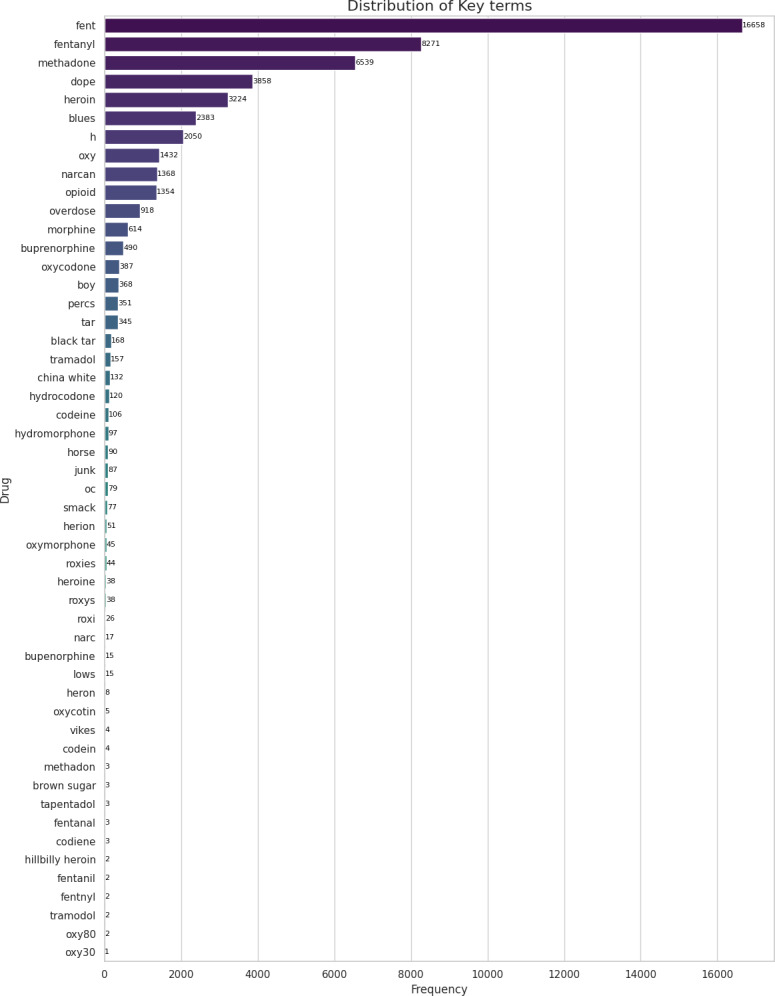
Distribution of opioid-related keywords in a sample of 80,000 Reddit posts.

To ensure that the collected posts were relevant to the topic of opioid overdose, we implemented a keyword-based filtering system. We used a comprehensive set of nearly 50 carefully selected keywords, as shown in Figure 1, along with their word frequencies. These include formal terms such as “overdose,” “opioid,” “fentanyl,” “heroin,” “OxyContin,” and “Narcan,” as well as common slang and misspellings frequently encountered in social media discussions. Examples of slang and variations include “oxy,” “narc,” “fent,” “dope,” “smack,” as well as misspellings such as “herin,” “codein,” “codiene,” “herioin,” “percacet,” “fentnil,” “fentnyl,” “oxycontn,” “oxocodien,” and “narcan.” This inclusive keyword list helped capture informal and colloquial references to opioids, reflecting the informal nature of Reddit conversations. Our keyword filtering also captured posts where opioid terms were used metaphorically, in usernames, or in unrelated contexts. These were manually labeled as unrelated during annotation to ensure dataset accuracy. Examples include phrases such as “@codeine_XXXX,” “I am addicted to my X like fentanyl,” or “Yoga and maple brown sugar roasted butternut squash.” This step prevented irrelevant content from affecting the analysis.

As social media platforms such as Reddit host multilingual communities, we applied language filtering to include only posts written in English. To ensure consistency and simplify analysis, we used a Python language detection library (eg, langdetect) to automatically identify and retain only English-language posts, excluding all non-English content from the dataset. This step ensured that the dataset accurately reflects English discussions relevant to our study.

**Figure 2.**
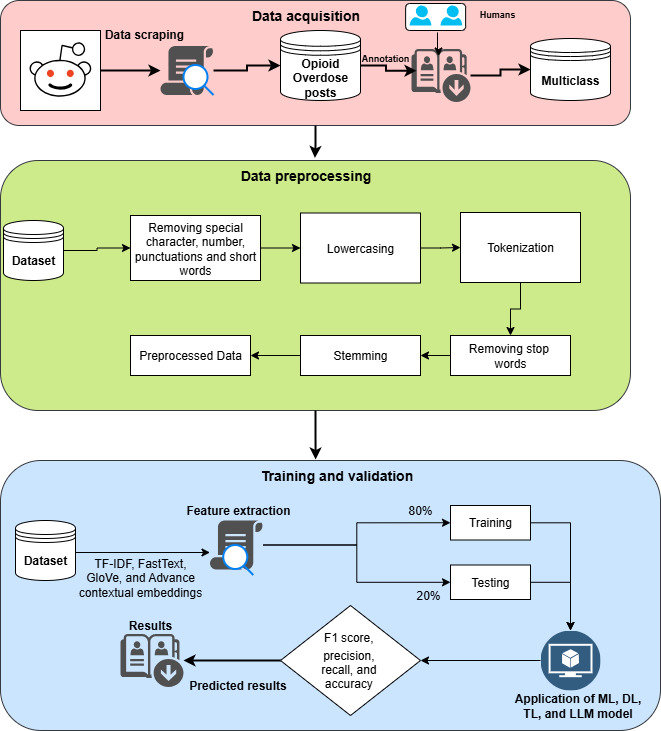
. Proposed methodology and design. DL: deep learning; LLM: large language model; ML: machine learning; TF-IDF: term frequency–inverse document frequency; TL: transfer learning.

After the construction of the dataset, we used some preprocessing steps to clean and standardize the text data for improved model performance. As our dataset was related to opioid overdose discussions extracted from Reddit, it contained a lot of informal, noisy, and user-generated content that needed careful handling. Initially, we removed hashtags, emojis, and any embedded links to eliminate distractions and irrelevant symbols that do not contribute meaningfully to the actual content or context of the discussion. The next phase involved converting all text to lowercase to ensure uniformity, followed by expanding short or informal words into their full forms using the Python contractions library for standard English contractions (eg, “can’t” → “cannot,” “won’t” → “will not”). Finally, we removed posts with fewer than 15 characters, as they typically lacked meaningful information and could introduce noise into the analysis. These steps were crucial for ensuring the dataset was clean, consistent, and suitable for effective NLP, especially given the sensitive and critical nature of opioid-related discussions. Figure 2 presents the overall workflow of the proposed methodology.

### Annotation

Annotation is the procedure of classifying data into predefined and meaningful information for supervised learning models. In our dataset, annotation involves labeling textual data into predefined binary classes that represent different aspects of opioid-related conversations. This helps machine learning models understand hidden patterns in drug abuse, detect trends, and train models for automated classification and analysis.

### Data Annotation Guidelines

Following cleaning, the data were subjected to a rigorous manual annotation process. We defined 4 clear categories to label each post:

- Self-misuse: posts describing the Reddit opioid users’ use of the drugs or overdose experiences by self or may include confessions, health complaints, withdrawal symptoms, or stories of addiction
- External misuse: posts discussing overdose or opioid use experienced by others (eg, friends, family, institutions, or social groups) or such as news, community issues, or government/policy critique
- Information: posts sharing factual data, advice, news articles, or general information about opioids and overdose or includes posts by health professionals, nongovernmental organizations, or automated bots sharing links or advice
- Not related: posts that mention keywords but are unrelated to opioid overdose

### Annotation Process

In this study, the dataset was collaboratively created by the corresponding author with the assistance of 2 domain experts—PhD students in computer science—who had previously labeled the dataset. Their prior work on this dataset has been published, and they possess extensive experience in annotation within the opioid domain [34].

Initially, a subset of 500 Reddit posts was randomly selected for a pilot annotation phase to ensure a representative sample of opioid-related discussions. These posts were reviewed in collaboration with experts in addiction medicine to develop and refine the annotation guidelines. Each post was categorized into 1 of 4 predefined classes: self-misuse, external misuse, informational post, and not related (see section Data Annotation Guidelines). Table 1 provides examples for each category to illustrate the annotation criteria and support reproducibility. During this phase, all 3 annotators independently labeled the 500 posts to assess labeling quality and resolve discrepancies through discussion and majority voting. This iterative process facilitated refinement of the guidelines for improved clarity and consistency. After consensus on the guidelines, the remaining posts—bringing the total annotated dataset to 5046 posts—were labeled independently by all 3 annotators, following the finalized guidelines to maintain consistency across the dataset.

**Table 1.** Examples of opioid overdose taken from the dataset.

Reddit post	Class
Took way too many oxycodone tonight... feeling dizzy, can’t breathe properly. My chest is so heavy. Should I be worried? #opioids #help	Self-misuse
My cousin keeps overdosing on fentanyl, but he refuses to go to rehab. I’m scared for him.	External misuse
Opioid overdoses are a major crisis. If you or someone you know is struggling, carry naloxone and seek help. Harm reduction saves lives! #EndOverdose	Information
She’s my heroin—one smile from her and I’m gone. No rehab can fix this kind of addiction, and I don’t even want to quit. #LoveAddiction	Unrelated

Interannotator agreement was assessed using Cohen κ and achieved a strong score of 0.82 across the entire set of annotated posts, indicating high reliability among annotators. This rigorous annotation process ensured that the dataset was accurately labeled and suitable for downstream classification tasks.

### Corpus Characteristics and Standardization

Table 2 presents the label distribution in our annotated dataset before and after data augmentation. Initially, the dataset was imbalanced, with self-misuse having 1877 instances, whereas external misuse (1375 instances), information (1391 instances), and unrelated (403 instances) were underrepresented, resulting in a total of 5046 samples. To address this imbalance, we applied back-translation to augment the minority classes. Back-translation is a widely used technique in NLP [50,51] that enhances dataset diversity by translating text into an intermediate language and then translating it back to the original language. This process generates lexically and syntactically varied samples while preserving the original semantic meaning. Unlike simple duplication, back-translation introduces natural linguistic variation, helping the model generalize better. After augmentation, each class was expanded to contain 1877 instances, leading to a balanced dataset of 7508 samples, which supports more stable and unbiased model training.

**Table 2.** Label distribution of the proposed dataset before and after data augmentation.

Label	Before augmentation	After augmentation
External misuse	1375	1877
Information	1391	1877
Self-misuse	1877	1877
Unrelated	403	1877
Total samples	5046	7508

Table 3 provides a comprehensive overview of the linguistic characteristics of our final dataset after data augmentation, which consists of 7508 posts. The dataset contains a rich and diverse vocabulary of 25,993 unique words, reflecting the variety of expressions used across different user-generated texts. In total, the dataset comprises over 2.76 million characters, 603,107 words, and 23,615 sentences, indicating the substantial size and depth of the corpus. On average, each sentence contains approximately 17.68 words, which suggests moderately complex sentence structures, whereas each post contains about 5.23 words, highlighting the brevity typical of social media posts similar to those on Reddit. This combination of compact posts with meaningful linguistic diversity makes the dataset both challenging and valuable for training robust NLP models. Multimedia Appendix 1 shows the word cloud of our dataset.

**Table 3.** Dataset statistics.

Metric	Value
Post	7508
Vocabulary	25,993
Total characters	2,762,238
Total word	603,107
Total sentence	23,615
Average words per sentence	17.679
Average words per post	5.23

### Application of Models Training and Testing

**Figure 3.**
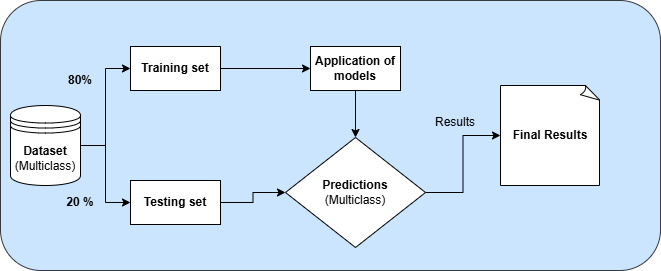
Application of models training and testing phase.

In this section, we explain the application of the models during the training and testing phases. Figure 3 presents a detailed pipeline for a multiclass classification task using a variety of machine learning, deep learning, transformer-based models, and LLMs. The process begins with a multiclass dataset, which is divided into 2 parts: 80% of the data are allocated for training the models, whereas 20% are held out as an independent testing set for evaluating performance. This 80‐20 split was consistently applied across all models to ensure uniformity in data handling.

The training phase involves applying multiple types of models across 4 main categories. In traditional machine learning, models such as RF, XGB, SVM, and LR are trained using TF-IDF features to capture term importance. For deep learning, architectures such as CNN and BiLSTM are used, supported with pretrained word embeddings, such as FastText and GloVe, to better capture semantic relationships in text. The pipeline also incorporates powerful transformer-based models, including XLM-R, DistilBERT, mBERT, and Bio_ClinicalBERT, all of which are fine-tuned to adapt to the dataset. These transformer-based models leverage contextual understanding from large-scale corpora. In addition, an LLM, GPT-3.5-turbo-0125, is fine-tuned (Table 2), using its advanced natural language understanding capabilities. LLMs generate high-quality contextual embeddings that capture deep semantic and syntactic information, enabling a more accurate and nuanced understanding of language compared to traditional embedding methods.

Once all models are trained, they are evaluated on the held-out testing set, generating multiclass predictions. These predictions are then compiled and analyzed to assess accuracy, precision, recall, and *F*_1_-scores. The results from each category of models help determine which methods are most effective for the task. This comprehensive approach enables a comparative analysis across model types.

Owing to the significant computational resources and financial costs associated with fine-tuning LLMs such as GPT-3.5, cross-validation or separate validation sets were not feasible. Instead, hyperparameter tuning and model selection were conducted exclusively on the training data, whereas the held-out test set was strictly reserved for final unbiased evaluation.

In the final stage, the top-performing model was deployed on an unlabeled dataset comprising 74,975 additional Reddit posts. This enabled a detailed temporal analysis of opioid-related discussions over the 6-year period, uncovering trends and shifts in public perception, self-reported use, and information sharing around opioid drugs.

Table 4 summarizes the hyperparameter tuning settings applied during model training for each learning approach, such as LLM, transformers, machine learning, and deep learning—in the context of a multiclass classification task on opioid overdose data. Hyperparameter tuning was conducted using grid search exclusively on the training set, following an 80% to 20% train-test split, as detailed in the model training section. No separate validation set was used; instead, all parameters were optimized within the training partition to avoid data leakage and ensure generalization. For the LLM, GPT-3.5-turbo-0125 was tuned with parameters such as epochs (3), batch size (12), learning rate (2), seed (1278387729), and processed 1,761,342 tokens, reflecting the scale and reproducibility of fine-tuning. The transformer models, including mBERT, Bio_ClinicalBERT, DistilBERT-base-uncased, and XLM-R, were optimized using a learning rate of 2e-5, 3 epochs, batch size of 32, and trained with the AdamW optimizer and CrossEntropyLoss, a typical setup for classification tasks using pretrained language models. The machine learning models had distinct tuning schemes: SVM used random state (42), kernels (linear and RBF), C (1.0), and gamma (auto); XGB was tuned with 100 estimators, max depth (6), and learning rate (0.3); RF used standard parameters such as n_estimators (100), max_depth (none), and bootstrap (true) to control the number and depth of trees and sampling behavior; and LR used random state (42), max iterations (1000), C value (0.1), and the liblinear solver. Finally, the deep learning models (BiLSTM and CNN) were trained using a learning rate of 0.1, 5 epochs, an embedding dimension of 300, and a batch size of 32, suitable for word embedding–based architectures. These hyperparameter settings were selected through grid search to systematically explore model performance variations.

**Table 4.** Optimum values identified for the hyperparameters of the proposed model.

Learning approach and models		Hyperparameter	Grid search
Large language model			
GPT-3.5-turbo-0125		epochs, batch size, LR^a^, seed, trained tokens	3, 12, 2, 1278387729, 1761342
Transformer			
mBERT^b^, bert-based-uncased, distelbert-based-uncased, XLM-R^c^		learning rate, epoch, batch size, Optimizer, Loss Function	2e-5, 3, 32, AdamW, CrossEntropyLoss
Machine learning			
SVM^d^		random state, kernel, c value, gamma	42, linear and rbf, 1.0, auto
XGB^e^		n_estimators, max_depth, learning_rate	100, 6, 0.3
RF^f^		n_estimators , max_depth	100, none
LR		random state, max_iter, c value, solver	42, 1000, 0.1, liblinear
Deep learning			
BiLSTM^g^ and CNN^h^		learning rate, epoch, embedding_dim, batch size	0.1, 5, 300, 32

a LR: logistic regression

b mBERT: multilingual bidirectional encoder representations from transformers.

c XLM-R: XLM-RoBERTa.

d SVM: support vector machine.

e XGB: Extreme Gradient Boosting.

f RF: random forest.

g BiLSTM: bidirectional long short-term memory.

h CNN: convolutional neural network.

### Ethical Considerations

This study involved the creation of a novel annotated dataset using publicly available text posts from Reddit. Only content that was freely accessible without any login or private access was collected, and no interaction with users occurred. All data were processed in accordance with Reddit’s terms of service, and personally identifiable information was removed before annotation. As the study used publicly available, nonidentifiable content and presented results in aggregate, institutional review board or equivalent ethics approval was not required.

## Results

### Overview

In this section, we present the experimental results obtained using machine learning, deep learning, transformer-based models, and LLMs, following the methodology outlined in the previous section.

### Machine Learning Results

We used several machine learning models to get a full picture of how diverse algorithms performed on our dataset. Owing to the various strengths and assumptions of individual models, the set of varied models will help in drawing a better and trustworthy analysis. This comparative approach helps identify which model performs best for our specific task and ensures that the results are not biased toward a single algorithm’s limitations.

Table 5 presents the performance of 4 different machine learning models—such as LR, RF, SVM, and XGB—applied to a multiclass classification task on opioid-related social media chatter using TF-IDF. TF-IDF was used as a feature extraction technique to convert unstructured text into numerical features that machine learning models can understand and process. It helps highlight words that are important and meaningful in a specific document while minimizing the influence of commonly used words that may not carry significant context. The task involves categorizing Reddit chatter posts into 4 distinct classes: self-misuse, external misuse, informational content, and unrelated posts. Each model’s effectiveness is measured using 4 standard metrics: precision, recall, *F*_1_-score, and accuracy. Among the models, the RF achieved the highest performance across all metrics (0.85), indicating superior capability in correctly identifying various opioid-related classes in the dataset. XGB followed closely with scores of 0.83, suggesting it also performed well in handling the complexity of multiclass distinctions in social media text. SVM and LR trailed behind, with SVM scoring 0.79 and LR the lowest at 0.77 across all metrics. This evaluation suggests that ensemble models such as RF and XGB are more effective in understanding and classifying nuanced opioid-related discourse on social platforms compared to linear and margin-based methods.

**Table 5.** Results for machine learning models.

Model	Precision	Recall	*F*_1_-score	Accuracy
LR^a^	0.77	0.77	0.77	0.77
RF^b^	0.85	0.85	0.85	0.85
SVM^c^	0.79	0.79	0.79	0.79
XGB^d^	0.83	0.83	0.83	0.83

a LR: logistic regression.

b RF: random forest.

c SVM: support vector machine.

d XGB: Extreme Gradient Boosting.

### Deep Learning Results

Table 6 compares the performance of 2 popular deep learning models—such as CNN and BiLSTM—using 2 different pretrained word embedding techniques, FastText [52] and GloVe [53], for classifying opioid-related social media chatter for the classification of opioid-related Reddit posts into 4 categories, including self-misuse, external misuse, informational content, and unrelated posts. We used these word embeddings to capture the semantic meaning of words based on their usage in large corpora, allowing models to understand the context and relationships between words more effectively. When using FastText embeddings, the CNN model outperforms BiLSTM, achieving the highest scores across all metrics with a precision of 0.82, recall of 0.80, *F*_1_-score of 0.79, and accuracy of 0.80. In contrast, the BiLSTM underperforms with slightly lower values. With GloVe embeddings, both CNN and BiLSTM perform similarly, with CNN achieving a slight edge in recall and accuracy (both 0.79) compared to BiLSTM (0.78). Overall, FastText embeddings combined with CNN yielded the best performance, suggesting that FastText’s subword-level information is more effective in capturing linguistic nuances in opioid-related discussions, especially when processed with CNN’s ability to extract local patterns in text.

**Table 6.** Results for deep learning models.

Model		Precision	Recall	*F*_1_-score	Accuracy
FastText					
CNN^a^		0.82	0.8	0.79	0.8
BiLSTM^b^		0.77	0.76	0.76	0.76
GloVe					
CNN		0.79	0.79	0.78	0.79
BiLSTM		0.79	0.78	0.78	0.78

a CNN: convolutional neural network.

b BiLSTM: bidirectional long short-term memory.

### Transformer Results

We have used transformer models, as they better comprehend the meaning and contextual relationships of words within a sentence compared to the models used in the previous sections, such as machine learning and deep learning approaches. Their ability to capture complex dependencies within the text contributes to improved performance, particularly in the classification of challenging and nuanced textual data [54].

Table 7 evaluates transformer-based language models—XLM-R, DistilBERT, mBERT, and Bio_ClinicalBERT—on their performance in classifying opioid-related social media chatter in a multiclass scenario. Among these, XLM-R and mBERT deliver the top results, each achieving a high *F*_1_-score and accuracy of 0.89, with XLM-R slightly leading in precision (0.90). These results highlight their strong capability in handling multilingual or diverse language patterns often found in social media discussions. DistilBERT and Bio_ClinicalBERT also perform well, scoring 0.88 across all metrics, but fall just short of the leading models. Interestingly, despite Bio_ClinicalBERT being pretrained on clinical texts, it does not outperform general-purpose multilingual models in this context, possibly due to the informal, varied nature of social media language. Overall, XLM-R and mBERT emerge as the most effective models for understanding and classifying opioid-related content in a multilingual, user-generated text environment.

**Table 7.** Result for transformer models.

Model	Precision	Recall	*F*_1_-score	Accuracy
XLM-R^a^	0.9	0.89	0.89	0.89
DistilBert	0.89	0.88	0.88	0.88
mBERT^b^	0.89	0.89	0.89	0.89
Bio_ClinicalBERT	0.88	0.88	0.88	0.88

a XLM-R: XLM-RoBERTa.

b mBERT: multilingual bidirectional encoder representations from transformers.

### LLM Results

Table 8 presents how well GPT-3.5 Turbo performed on a multiclass classification task focused on opioid-related social media chatter, with categories such as self-misuse, external misuse, information, and not related. On the training set, the model scored almost perfectly with 97% precision, recall, *F*_1_-score, and accuracy, meaning it could accurately distinguish between people talking about their own misuse, others’ misuse, sharing informational content, or unrelated topics. When tested on unseen data, it still performed strongly with 93% across all metrics, showing that it generalizes well and can reliably interpret real-world conversations without major overfitting. This strong performance indicates the model can effectively support tasks such as public health monitoring or early detection of opioid misuse trends by correctly categorizing social media discussions into these nuanced classes.

**Table 8.** GPT-3.5-turbo performance on multiclass opioid-related social media classification in training and test datasets.

Model	Precision	Recall	*F*_1_-score	Accuracy
XLM-R^a^	0.9	0.89	0.89	0.89
DistilBert	0.89	0.88	0.88	0.88
mBERT^b^	0.89	0.89	0.89	0.89
Bio_ClinicalBERT	0.88	0.88	0.88	0.88

a XLM-R: XLM-RoBERTa.

b mBERT: multilingual bidirectional encoder representations from transformers.

We used GPT-3.5 Turbo in this task because it is a powerful, general-purpose language model capable of understanding nuanced, context-rich data—such as informal and diverse social media posts [55]. Unlike traditional classifiers, GPT-3.5 Turbo can leverage deep semantic understanding and contextual reasoning, which is particularly useful for the ambiguous and unstructured language commonly found in online discussions about opioids.

### Temporal Analysis

**Figure 4.**
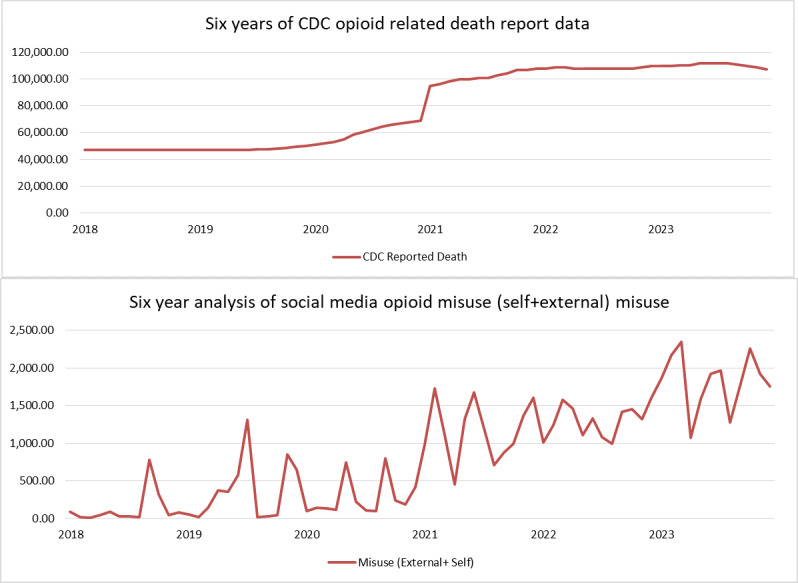
Trends in opioid-related CDC mortality and social media discourse from January 1, 2018, to December 30, 2023. CDC: Centers for Disease Control and Prevention.

Figure 4A presents the official mortality records over a 6-year period [56], whereas Figure 4B shows the volume of misuse-related posts (combined external and self) and informational posts related to substance use during the same time frame. To conduct the temporal analysis of opioid abuse, we trained our top-performing model (GPT-3.5) on 74,975 unlabeled posts, classifying them into 4 key categories: self-misuse, external misuse, information, and unrelated.

From 2018 to 2023, the temporal trends of Reddit misuse-related discussions show a striking alignment with the CDC’s opioid-related death statistics, suggesting that social media discourse mirrors real-world shifts in the opioid crisis. In the early years (2018‐2019), both misuse post volumes and CDC deaths remained comparatively lower and more stable, with occasional local spikes such as September and October 2018, where misuse posts surged to 780 and 324, respectively, despite steady death counts around 47,000. However, starting mid-2019, sharp increases in misuse chatter emerged, most notably in June (580 posts) and July (1310 posts), which preceded or coincided with gradual rises in reported deaths—indicating that online communities may detect or reflect growing misuse patterns ahead of official mortality escalation.

The year 2020 marks a turning point. Amid the COVID-19 pandemic, both misuse posts and CDC deaths began climbing in parallel, with significant peaks in May (750 misuse posts and ~58,640 deaths) and September (796 misuse posts and ~65,811 deaths). This synchronous growth continued into 2021, where both metrics reached unprecedented levels. February 2021 saw 1728 misuse posts alongside 96,118 deaths, followed by sustained high activity through the year, culminating in December with 1600 misuse posts and 107,573 deaths. Notably, the temporal curves for 2021 suggest a near one-to-one relationship—when misuse posts rose, deaths also trended upward, reinforcing the notion that digital discourse intensity is tightly coupled with public health outcomes.

From 2022 onward, the pattern persisted but with even higher baselines. Monthly misuse counts regularly exceeded 1000, with peaks such as March 2022 (1576 misuse posts and 108,604 deaths) and December 2022 (1600 misuse posts and 109,413 deaths). In 2023, the relationship became even clearer: February’s 2167 misuse posts coincided with 109,714 deaths, whereas March reached the dataset’s highest misuse level at 2345 posts alongside 110,084 deaths. Although CDC death counts plateaued toward late 2023, misuse posts remained elevated, suggesting sustained concern and conversation within online communities even when mortality rates slightly dipped.

To quantify this relationship, we calculated the Pearson correlation coefficient (*r*=0.854) and the corresponding *P*-value (*P*<.001). These results indicate a strong positive correlation between Reddit misuse posts and CDC death records, which is statistically highly significant, confirming that the observed alignment is unlikely due to chance.

Overall, the temporal comparison indicates that Reddit discussions—particularly misuse-related categories—tend to rise and fall in close alignment with CDC mortality trends, sometimes even leading them. This finding underscores the value of social media monitoring as a complementary, potentially more immediate signal for detecting shifts in the opioid crisis, enabling earlier interventions before official data fully reflects the magnitude of the problem.

### Error Analysis

Table 9 presents the results of the top-performing models in each learning approach. The RF model delivered the best results in the traditional machine learning setting since it reached 0.85 in precision, recall, *F*_1_-score, and accuracy. It is implied by its regular outcomes that RF is very efficient in recognizing the patterns in structured data on the basis of opioid-related discussions. RF has an ensemble nature that enables generalization, and therefore, it acts well in the case of an engineered dataset with moderate complexity.

**Table 9.** Top-performing models in each learning approach.

Model	Precision	Recall	*F*_1_-score	Accuracy
RF^a^	0.85	0.85	0.85	0.85
CNN^b^ (FastText)	0.82	0.8	0.79	0.8
XLM-R^c^	0.9	0.89	0.89	0.89
GPT-3.5	0.93	0.93	0.93	0.93

a RF: random forest.

b CNN: convolutional neural network.

c XLM-R: XLM-RoBERTa.

When using the deep learning models with FastText and GloVe, the CNN using the FastText embeddings proved most effective. The model achieved a highest *F*_1_-score of 0.79 and an accuracy of 0.80. This pairing demonstrates the importance of both word representation and architecture when processing short, varied user-generated content.

In transformer-based models, the XLM-R model performed the best with a score of 0.89 (*F*_1_-score) and accuracy of 0.89. It is also an appropriate tool to work with different language patterns on social media because of its multilingual training and well-developed contextual awareness. It performed better than other transformer models, including mBERT and DistilBERT, which means that it is strong in inferring subtle behavioral cues about the users talking about opioids.

GPT-3.5 Turbo demonstrated the best performance among all models, achieving a score of 0.93 across all evaluation metrics, including precision, recall, *F*_1_-score, and accuracy. This consistent and high performance highlights the model’s effectiveness in handling the classification task. The reason why this LLM is exemplary is that it has a deep sense of understanding, few-shot reasoning capacities, and generalization skills over complex, input-rich scenarios. The high performance indicates the increasing potential of generative models in supporting the real-world and multiclass classification problems that deal with such sensitive and subtle issues as the behavior of opioid use.

Table 10 provides a detailed breakdown of GPT-3.5 Turbo’s performance in classifying opioid-related social media posts into 4 distinct categories: external misuse, information, self-misuse, and unrelated on the training and testing datasets. These categories represent different contexts of opioid discussions: External misuse refers to posts about others misusing opioids, information includes educational or factual content, self-misuse captures individuals sharing their own misuse experiences, and unrelated denotes content not relevant to opioids. Each category was evaluated using standard metrics—precision, recall, and *F*_1_-score—along with support, which indicates the number of posts in each category.

**Table 10.** Class-wise score of the GPT-3.5-turbo model on training and test datasets.

Category		Precision	Recall	*F*_1_-score	Support
Test set					
External misuse		0.92	0.93	0.93	392
Information		0.91	0.92	0.91	391
Self-misuse		0.94	0.89	0.92	376
Unrelated		0.95	0.97	0.96	343
Training set					
External misuse		0.97	0.98	0.97	1506
Information		0.97	0.96	0.97	1498
Self-misuse		0.96	0.97	0.97	1502
Unrelated		0.99	0.97	0.98	1500

On the training set, GPT-3.5 Turbo demonstrated near-perfect performance, indicating strong learning of the patterns in the data. Precision and recall for most categories were extremely high, ranging from 0.96 to 0.99, and the *F*_1_-score was similarly impressive, with unrelated posts scoring the highest at 0.98. For example, external misuse, information, and self-misuse each achieved an *F*_1_-score of 0.97, reflecting the model’s ability to balance correctly identifying relevant posts while minimizing misclassification. These results suggest the model adapted well during training and could easily distinguish between nuanced content types.

On the test set, which reflects the model’s ability to generalize to unseen data, GPT-3.5 Turbo continued to perform strongly, although slightly lower than the training results—a normal and expected trend when models move from training to real-world scenarios. The *F*_1_-scores ranged from 0.91 to 0.96, indicating excellent generalization. Among the categories, unrelated posts were classified with the highest accuracy (*F*_1_-score=0.96), while self-misuse scored slightly lower (*F*_1_-score=0.92). This small dip may be because of the complexity and subtlety of personal language used in self-disclosure posts, which can be harder to interpret than factual or unrelated content. External misuse and information categories both performed well with *F*_1_-scores of 0.93 and 0.91, respectively, showing that the model handled context-based distinctions effectively.

**Figure 5.**
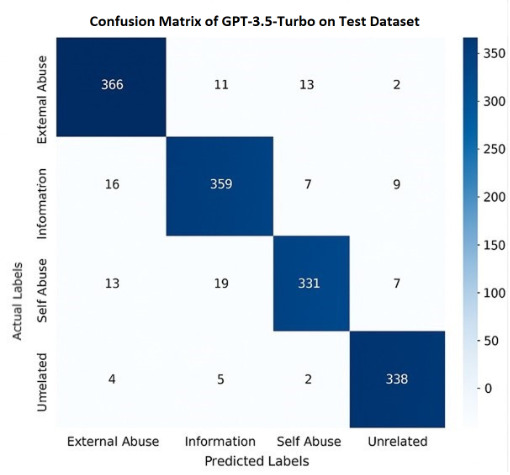
Confusion matrix of GPT-3.5-Turbo model on the test set.

**Figure 6.**
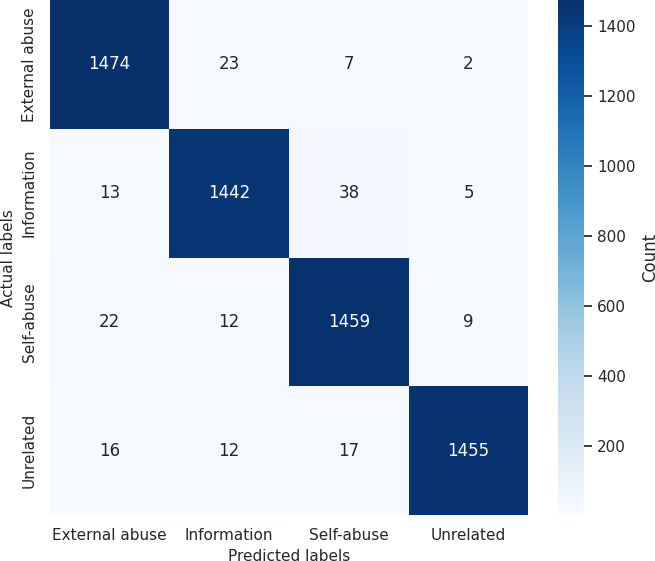
Confusion matrix of GPT-3.5-Turbo model on the training set.

Overall, these results highlight GPT-3.5 Turbo’s strength as an LLM in handling a sensitive and nuanced classification task. Its high precision and recall mean it can accurately detect opioid-related discussions while avoiding misclassifications, which is critical for real-world applications such as public health surveillance, early detection of misuse trends, and guiding harm reduction strategies. The slight drop from training to test performance confirms that the model is well-generalized without severe overfitting, making it a reliable tool for practical deployment in opioid-related content monitoring. We plotted the confusion matrices for both the training and testing sets. Figure 5 shows the confusion matrix for the test set, whereas Figure 6 shows the confusion matrix for the training set. The confusion matrix is a performance evaluation tool used for classification models. It helps us understand how well the model is performing by showing the breakdown of correct and incorrect predictions across the various classes.

## Discussion

### Principal Findings

This study presents a comprehensive comparison of various machine learning, deep learning, transformer, and LLMs to classify opioid-related social media chatter into 4 behavioral categories: self-misuse, external misuse, informational, and unrelated. Among traditional models, RF emerged as the best performer, achieving an *F*_1_-score of 0.85. In deep learning, the CNN model using FastText embeddings outperformed other configurations with an *F*_1_-score of 0.79. Transformer-based models, particularly XLM-R, delivered high accuracy and contextual understanding with an *F*_1_-score of 0.89. However, the GPT-3.5 Turbo model demonstrated the best overall performance across all learning approaches, achieving a perfect balance across all metrics (0.93), likely because of the balanced nature of the dataset and its advanced contextual reasoning abilities. The class-wise analysis further confirmed that GPT-3.5 Turbo is highly effective across all categories, with particularly strong performance in identifying unrelated and external misuse content. These findings highlight the increasing value of LLMs in toxicovigilance and behavior monitoring on social media platforms, particularly in sensitive domains such as opioid use.

### Comparison to Prior Work

While prior research, such as that by Webster et al [36], Dekeseredy et al [37], and Jaafar [41], has provided critical qualitative insights into media portrayals, stigmatizing narratives, and public discourse on opioid use, these studies often relied on manual analysis and lacked scalable frameworks for real-time monitoring. Some recent efforts used unsupervised methods to analyze opioid-related posts; however, they faced challenges such as noise, irrelevant content, and limited interpretability. In contrast, this study fills a critical gap by implementing a robust, supervised NLP pipeline, combining the power of pretrained embeddings and LLMs to enhance classification accuracy. Compared to earlier works that offered descriptive insights or small-scale automated models, the use of GPT-3.5 Turbo and transformer architectures in this study significantly improves the granularity, scalability, and reliability of opioid discourse classification. Furthermore, the inclusion of temporal analysis distinguishes this work by allowing researchers and policymakers to track behavior and crisis trends over time, an area not sufficiently addressed in previous literature.

### Strengths and Limitations

One of the key strengths of this study is its comprehensive evaluation across a broad range of models, from traditional machine learning to state-of-the-art LLMs. The balanced, manually annotated dataset, developed in collaboration with domain experts, provides a reliable foundation for multiclass classification and ensures consistency in labeling. The integration of temporal analysis using Reddit posts from 6 years further enhances the utility of this system for real-time public health surveillance. Moreover, the use of GPT-3.5 Turbo demonstrates the transformative potential of LLMs in extracting behavioral insights from noisy, user-generated data.

However, several limitations remain. First, the dataset, while diverse and balanced, is limited to Reddit and may not capture the full spectrum of discourse across other platforms such as Facebook, X, or TikTok. Second, while the dataset was perfectly balanced across classes, real-world social media data are often highly imbalanced, with many more “unrelated” posts than posts in other categories. This balancing decision improves model performance on all classes but may reduce generalizability to broader, naturally imbalanced datasets. Third, the model assigns only a single label per post, whereas in reality, a post may contain elements of multiple categories, for example, “I’ve been taking more oxycodone than my doctor prescribed because the pain is still bad, but I also read about overdose risks and want to warn others to be careful.” This single post includes aspects of self-misuse, external misuse, and informational content. When such posts are restricted to 1 label, important nuances may be lost. As a result, some nuances of user behavior may be lost when posts are forced into a single category.

Fourth, this study does not incorporate advanced geolocation techniques, such as user profile analysis and language-geography inference, which could enable more precise identification of posts originating from the United States—the primary country of interest in this study—as well as broader regional analysis. Integrating these techniques in future work may provide deeper insights into the geographical distribution of opioid-related discourse. Fifth, the model’s slightly lower recall in the self-misuse category suggests a need for more nuanced linguistic and contextual modeling in this area. In addition, although LLMs such as GPT-3.5 are powerful, they are computationally expensive and require significant resources to fine-tune or deploy at scale. Finally, while the model performs well in classification, it does not yet explain the underlying drivers or sentiment trends behind opioid behavior—areas that could be explored in future work.

### Conclusions and Future Work

This study demonstrates the significant potential of social media platforms, particularly Reddit, as valuable sources for real-time opioid surveillance through NLP and advanced machine learning techniques. By leveraging a large-scale annotated dataset and applying a range of supervised and transformer-based models, including fine-tuned LLMs, we classified opioid-related discourse into meaningful categories and uncovered temporal trends that align with known patterns in the ongoing opioid crisis. Our proposed supervised model, trained on manually annotated Reddit data, outperformed traditional unsupervised and baseline transformer models, achieving a classification accuracy of 0.93—a 9.16% improvement over classical machine learning model (RF 0.85). These results underscore the effectiveness of supervised learning, particularly when coupled with high-quality annotation and domain-specific preprocessing, in handling noisy and unstructured social media text. Moreover, the temporal analysis of 6 years of opioid-related chatter highlighted evolving patterns of public engagement, including spikes likely influenced by media coverage, policy changes, and societal events. Correlation analysis showed that misuse-related posts were strongly associated with CDC-reported deaths (Pearson *r*=0.854, *P*<.001), indicating that social media activity closely mirrors real-world mortality trends.

Although our proposed system has demonstrated strong performance in classifying opioid-related discussions and capturing temporal trends, several avenues remain for future exploration. First, expanding the analysis to include additional social media platforms, such as X (formerly Twitter), YouTube, and Facebook, could provide a more comprehensive view of online opioid-related discourse. Second, incorporating advanced geolocation techniques, such as user profile analysis and language-geography inference, could enable more precise country-specific and regional analysis, offering deeper insights into the geographical distribution of opioid-related discussions. Third, incorporating multimodal data—such as images, videos, and audio—may further enhance the system’s ability to detect signs of substance abuse. Third, improving the model’s ability to handle code-mixed and multilingual data will be essential for capturing opioid-related discussions in diverse linguistic communities. In addition, integrating geolocation inference techniques could support finer-grained spatial analyses, helping to identify regional hotspots in real time. Moreover, to further improve the robustness and generalizability of the models, we plan to expand and diversify our dataset to include more varied samples. This expansion will facilitate the implementation of more rigorous evaluation methods, such as cross-validation, which will help enhance the reliability and unbiased assessment of model performance.

## Supplementary material

10.2196/77279 Multimedia Appendix 1 Word cloud of keywords extracted from the dataset.

## Abbreviations

BiLSTM: bidirectional long short-term memory
CDC: Centers for Disease Control and Prevention
CNN: convolutional neural network
LLM: large language model
LR: logistic regression
mBERT: multilingual bidirectional encoder representations from transformers
NLP: natural language processing
NMPDU: nonmedical prescription drug use
RF: random forest
SVM: support vector machine
XGB: Extreme Gradient Boosting
XLM-R: XLM-RoBERTa
