# Supplementary figures and images for: Monitoring Opioid-Related Social Media Chatter Using Natural Language Processing and Large Language Models: Temporal Analysis
Source: JMIR Infodemiology. 2025 Nov 4;5:e77279. doi: 10.2196/77279 (PMC12585000; doi:10.2196/77279)

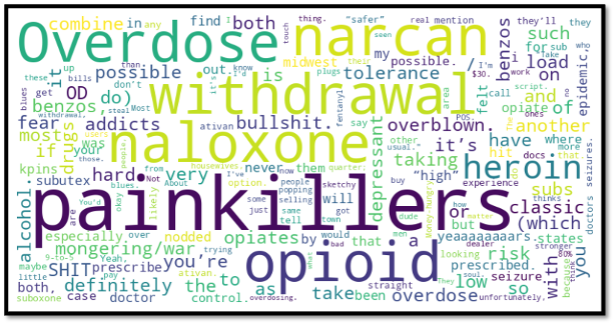

Supplement: Multimedia Appendix 1 [file infodemiology-v5-e77279-s001.png]
